# Switch from ambient to focal processing mode explains the dynamics of free viewing eye movements

**DOI:** 10.1038/s41598-017-01076-w

**Published:** 2017-04-24

**Authors:** Junji Ito, Yukako Yamane, Mika Suzuki, Pedro Maldonado, Ichiro Fujita, Hiroshi Tamura, Sonja Grün

**Affiliations:** 1 Institute of Neuroscience and Medicine (INM-6) and Institute for Advanced Simulation (IAS-6) and JARA BRAIN Institute I, Jülich Research Centre, Jülich, Germany; 2 Graduate School of Frontier Biosciences, Osaka University, Osaka, Japan; 3 Center for Information and Neural Networks, Osaka University and National Institute of Information and Communications Technology, Osaka, Japan; 4 BNI, CENEM and Programa de Fisiología y Biofísica, ICBM, Facultad de Medicina, Universidad de Chile, Santiago, Chile; 5 Theoretical Systems Neurobiology, RWTH Aachen University, Aachen, Germany

## Abstract

Previous studies have reported that humans employ ambient and focal modes of visual exploration while they freely view natural scenes. These two modes have been characterized based on eye movement parameters such as saccade amplitude and fixation duration, but not by any visual features of the viewed scenes. Here we propose a new characterization of eye movements during free viewing based on how eyes are moved from and to objects in a visual scene. We applied this characterization to data obtained from freely-viewing macaque monkeys. We show that the analysis based on this characterization gives a direct indication of a behavioral shift from ambient to focal processing mode along the course of free viewing exploration. We further propose a stochastic model of saccade sequence generation incorporating a switch between the two processing modes, which quantitatively reproduces the behavioral features observed in the data.

## Introduction

When we visually inspect our surroundings, we typically shift our gaze with saccadic eye movements^1^. Between saccades, there are periods of visual fixations, during which we sample visual information from the environment. Because only a tiny fraction of the visual field is projected onto the foveal region of the retina, where visual acuity is highest, the amplitude and direction of a saccade determine which part of the visual field is examined at the forthcoming fixation. Thus, how the eyes are moved by saccades has a crucial impact on the sampling and processing of information from the visual environment. Hence, the mechanism underlying saccade target selection has been a matter of great interest in vision science^2, 3^.

A body of studies have attempted to explain the mechanism of saccade target selection in terms of local features (e.g., contrast, color, orientation, etc.) and global configurations (e.g., scene gist, object congruency, etc.) of visual scenes (see ref. 3 for an extensive review). These studies mostly focus on deriving a map that predicts gaze-attracting regions in a given scene (saliency map), thereby paying less attention to the dynamics of saccade target selection, i.e., in what order different parts of the scene are scanned. Nonetheless, experimental studies of eye movements during free viewing of static scenes have reported a robust, systematic, and stimulus-independent tendency in the time course of saccade target selection. For instance, the amplitude of saccades tends to decrease over time along the course of free viewing^4, 6–9^. These studies also reported that this decrement in saccade amplitude is accompanied by an increment in fixation duration, confirming earlier observations reported in the pioneering work by Buswell^10^ on free viewing of pictorial images. These changes in saccade amplitude and fixation duration have been interpreted to reflect a shift of the visual processing mode from an initial ambient mode for grasping global configurations of the scene, characterized by the combination of large saccades and short fixations, to a subsequent focal mode for examining local features, characterized by the combination of small saccades and long fixations^5, 11^.

Interested in the neuronal mechanism underlying the dynamics of saccade target selection, we started neurophysiological recordings from freely viewing monkeys. However, as we critically examined eye movements of the monkeys, especially their relation to the ambient and focal processing modes, we identified at least two issues that need to be clarified before we can link the neuronal activity to eye movement behavior. First, while previous studies have characterized these processing modes only by the combination of saccade amplitude and fixation duration, there has been no consideration of the visual gaze position onto objects or background. We conjecture that the ambient and the focal processing modes would be more directly characterized by saccades across and within visual objects, respectively. Second, while previous studies postulated the two exploration modes and a shift from one mode to the other, there has been no systematic explanation about how and at what timing this shift occurs. The two modes were inferred from the relation of saccade amplitude and fixation duration, which only shows a gradual change over time and exhibits no clear boundary between the periods of the two modes. We conjecture that the shift between the two exploration modes occurs as an abrupt switch with variable switch timings across trials, still resulting in a smooth change of the eye movement parameters as observed in the previous studies after trial averaging.

Motivated by these interests, we conducted eye-tracking experiments in macaque monkeys freely viewing scenes composed of multiple complex visual objects. Here we report a quantitative study analyzing their eye movements focusing on whether individual eye movements are performed within an object or are across objects. Our results indicate that the monkeys perform ambient exploration across objects at the beginning of a free viewing trial followed by a focal examination of individual objects. We also propose a stochastic model of saccade sequence generation based on a switch between two distinct saccade generation modes. This model explains the observed dynamics of our free viewing eye movement data and also allows to infer the timing of the mode switch from the data.

## Results

**Figure 1:**
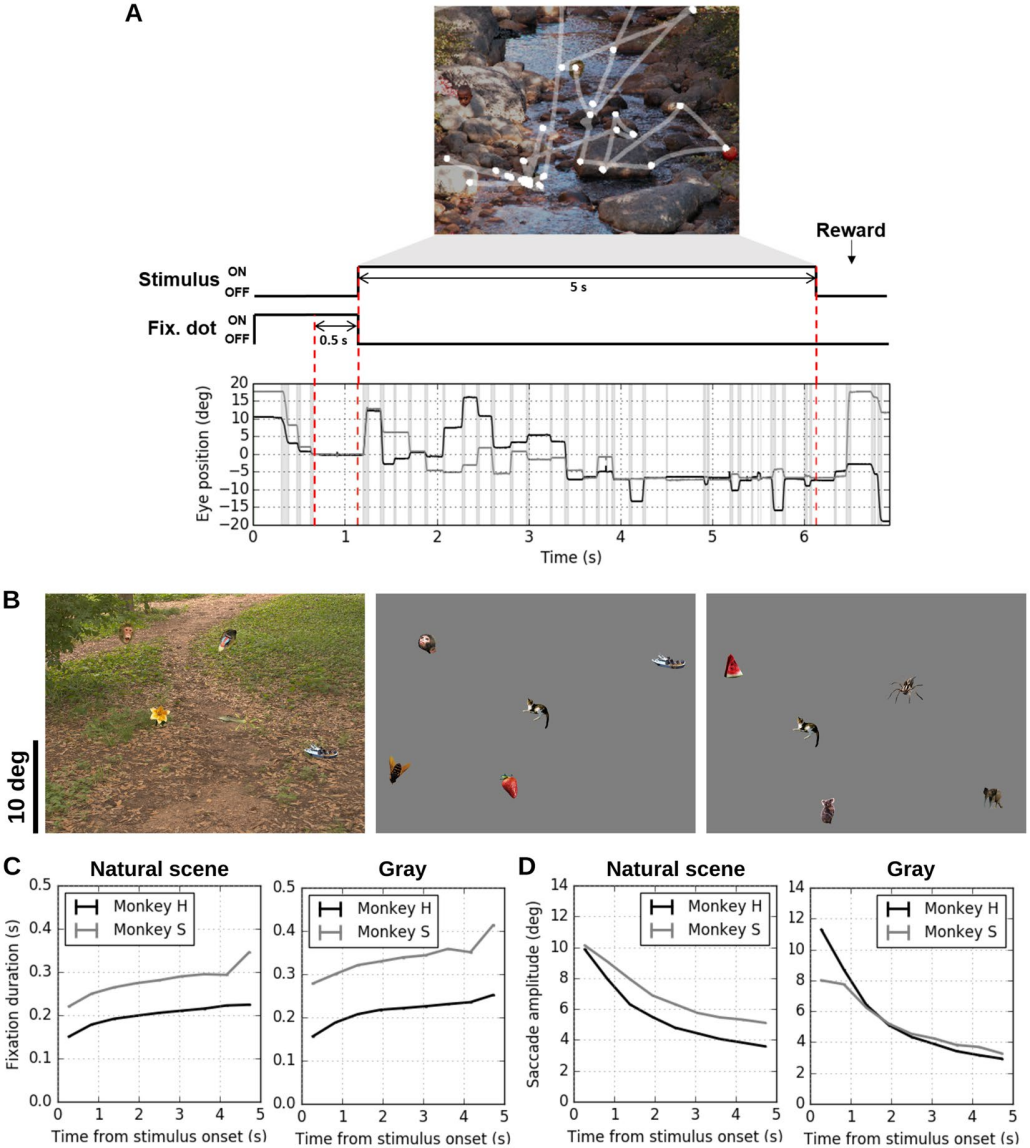
Free viewing experiment. (**A**) Behavioral paradigm. First, a gray background with a central fixation spot was presented. After the monkey had fixated for 0.5 s (for monkey H) or 0.3 s (for monkey S) on the fixation spot, the stimulus image was presented for five seconds. If the monkeys kept their gaze within the stimulus image, they got rewarded. The upper panel shows the gaze trajectory (white trace) during a representative free viewing trial performed by monkey H, overlaid on the actual stimulus image viewed in that trial. The bottom plot represents the time courses of horizontal (black) and vertical (gray) eye positions during the free viewing shown in the upper panel. Gray shades indicate the periods of saccades, between which are fixations. (**B**) Example stimulus images. In each stimulus image, five object images (2 deg in diameter, taken from the Microsoft image gallery; see Methods for details) are randomly placed on a natural scene image (left, taken from the natural image databases of University of Texas at Austin^42^ and McGill University^43^; see Methods for details) or a solid gray background (middle and right) subtending 34.8 × 26.1 deg^2^. The scale bar to the left indicates 10 deg. For all stimulus images used for monkey H, one of the five objects is intentionally placed at the center of the image (example: the middle panel), while the stimuli for monkey S do not have this constraint (example: the right panel). (**C**) Fixation duration as a function of viewing time. The mean fixation durations (line) and their standard errors (error bars, very small and almost invisible) were calculated at each time bin (bin size: 500 ms, no overlap between neighboring bins) for fixations of which onset times were within the corresponding bin. Left: stimulus images composed of natural scene background with five inserted objects. Right: gray background instead of natural scenes. (**D**) Saccade amplitude as a function of viewing time. The mean saccade amplitudes (line) and their standard errors (error bars) were calculated in the same manner as in (**C**). Left: natural scene backgrounds. Right: gray backgrounds.

Two macaque monkeys, referred to as monkey H and S hereafter, performed trials of free viewing of five seconds duration (Fig. 1A). Details of experimental methods are given in Methods section. Briefly, a trial was initiated by a 500 ms (for monkey H) or 300 ms (for monkey S) fixation at a central fixation point, and then a stimulus image for free viewing was presented. The monkeys were rewarded if they kept their gaze within the boundaries of the stimulus image for five seconds. The stimulus images for free viewing were composed by placing five object images (of about 2 degrees (deg) of visual angle in diameter) on natural scene images or a gray background (Fig. 1B; see Methods for further details of stimulus preparation). Throughout the free viewing trials the vertical and horizontal eye positions were recorded with a scleral search coil system. After the recording, eye events (fixations and saccades) were extracted offline based on eye velocity and acceleration criteria (see Methods for details). Monkey H performed 5,109 trials (over 25 recording sessions; one or two sessions per day), in which we identified 105,707 fixations and 106,052 saccades. Monkey S performed 7,472 trials over 79 sessions (one or two sessions per day), in which 101,439 fixations and 101,885 saccades were identified.

We first checked whether the saccades and fixations performed by the monkeys had the same temporal characteristics as observed in humans. Plots of saccade amplitudes and fixation durations as a function of trial time (Fig. 1C and D) clearly showed that both monkeys performed eye movements with increasing fixation durations and decreasing saccade amplitudes during a trial. These tendencies were observed irrespective of whether the stimulus backgrounds were natural scene images (Fig. 1C and D, left) or solid gray (Fig. 1E and F, right), indicating that complex natural scene backgrounds are not relevant for the temporal evolution of the eye event parameters as observed in human free viewing experiments^5, 6^. To simplify further analyses as well as to avoid parameters not relevant to the aspects considered here, we focused only on the data obtained from trials with gray background stimuli.

**Figure 2:**
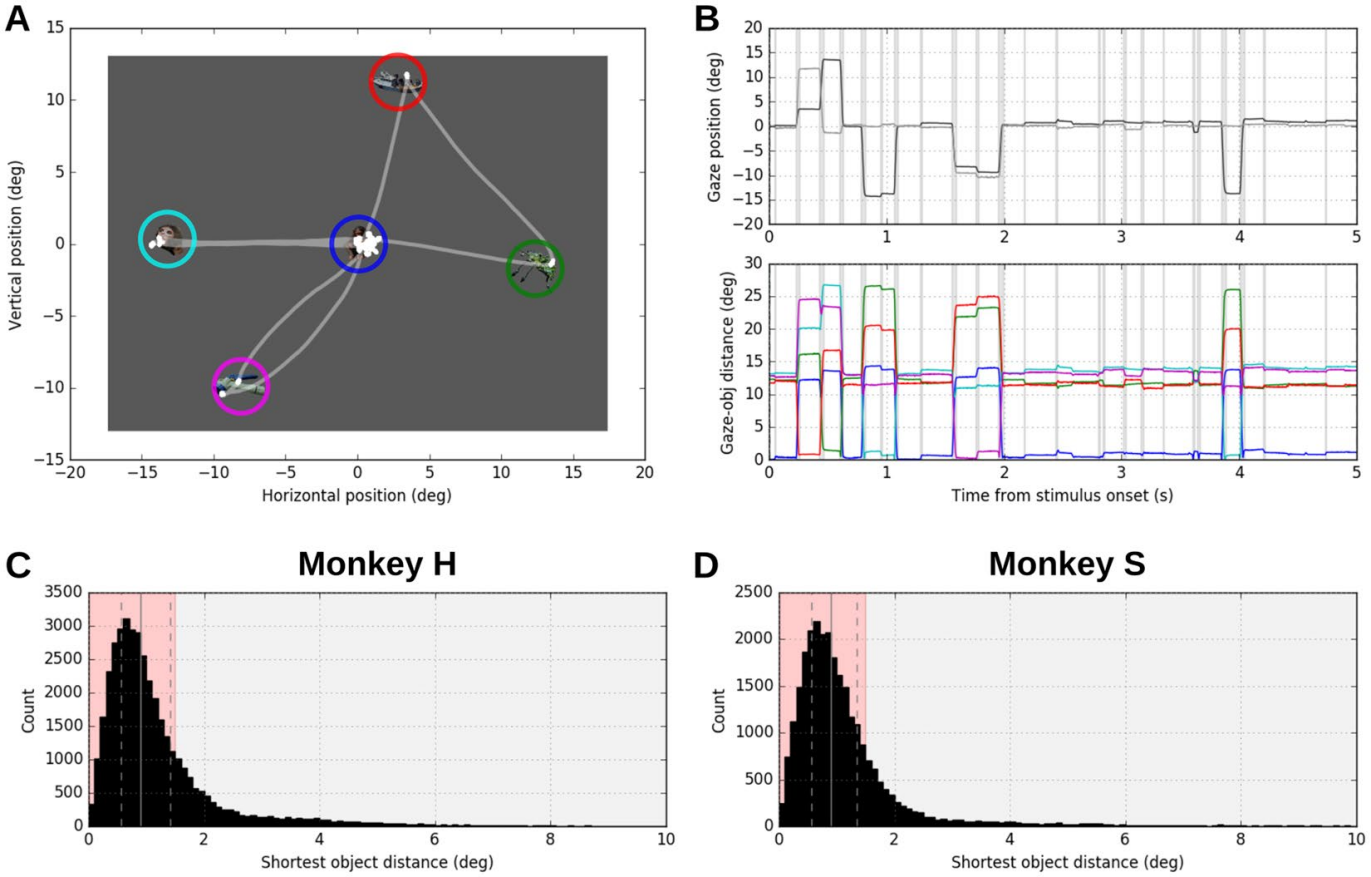
Definition of object fixation and background fixation. (**A**) Eye position trajectory from an example free viewing trial, overlaid on the stimulus image used in the trial. The circles marking objects are only for illustration purpose and were not visible to the monkeys. Object images are taken from the Microsoft image gallery. (**B**) Top: time courses of the horizontal (black) and vertical (gray) eye positions during the free viewing shown in (**A**). Bottom: the time courses of the distances from the instantaneous eye position to the center of 5 objects, plotted with different colors corresponding to the circles marking the objects in (**A**). Gray shades indicate the periods of saccades. (**C**,**D**) Distribution of the shortest object distance (the shortest of the five object distances of an individual fixation) computed from fixations performed by monkeys H (**C**) and S (**D**). Bin width is 0.1 deg. Solid and dashed vertical lines indicate the median and the 1st and 3rd quartiles, respectively. The red shaded area indicates the range of object fixations, defined as fixations of which shortest object distances were less than 1.5 deg.

Although we did not require the monkeys to explore the stimulus images, both monkeys spontaneously performed saccades across the stimuli with most of the fixations on and around the objects (Fig. 2A). The distance from a given fixation position to the center of the object closest to the fixation, termed here the *shortest object distance* of a fixation, was typically below two degrees (Fig. 2B). The distribution of the shortest object distances had similar shapes for the two monkeys: (1^st^ quartile, median, 3^rd^ quartile) = (0.562, 0.896, 1.417) deg for monkey H and (0.568, 0.892, 1.352) deg for monkey S (Fig. 2C and D). The fixations of which the shortest object distances were shorter than 1.5 degrees (roughly corresponding to the radius of the object images plus the size of foveal vision; see Methods for details) were considered as fixations on objects (termed *object fixations*), while all the others were considered as fixations on the background (termed *background fixations*). In both monkeys object fixations comprised about 80% of all fixations: 30,693 object fixations and 9,030 background fixations for monkey H; 27,427 object fixations and 5,511 background fixations for monkey S. These results confirm that the visual exploration performed by the monkeys was not a random wandering of the gaze position irrelevant to the structure of stimulus images but seems to indicate an intentional scanning of relevant features in the given image (e.g. Fig. 2A).

**Figure 3:**
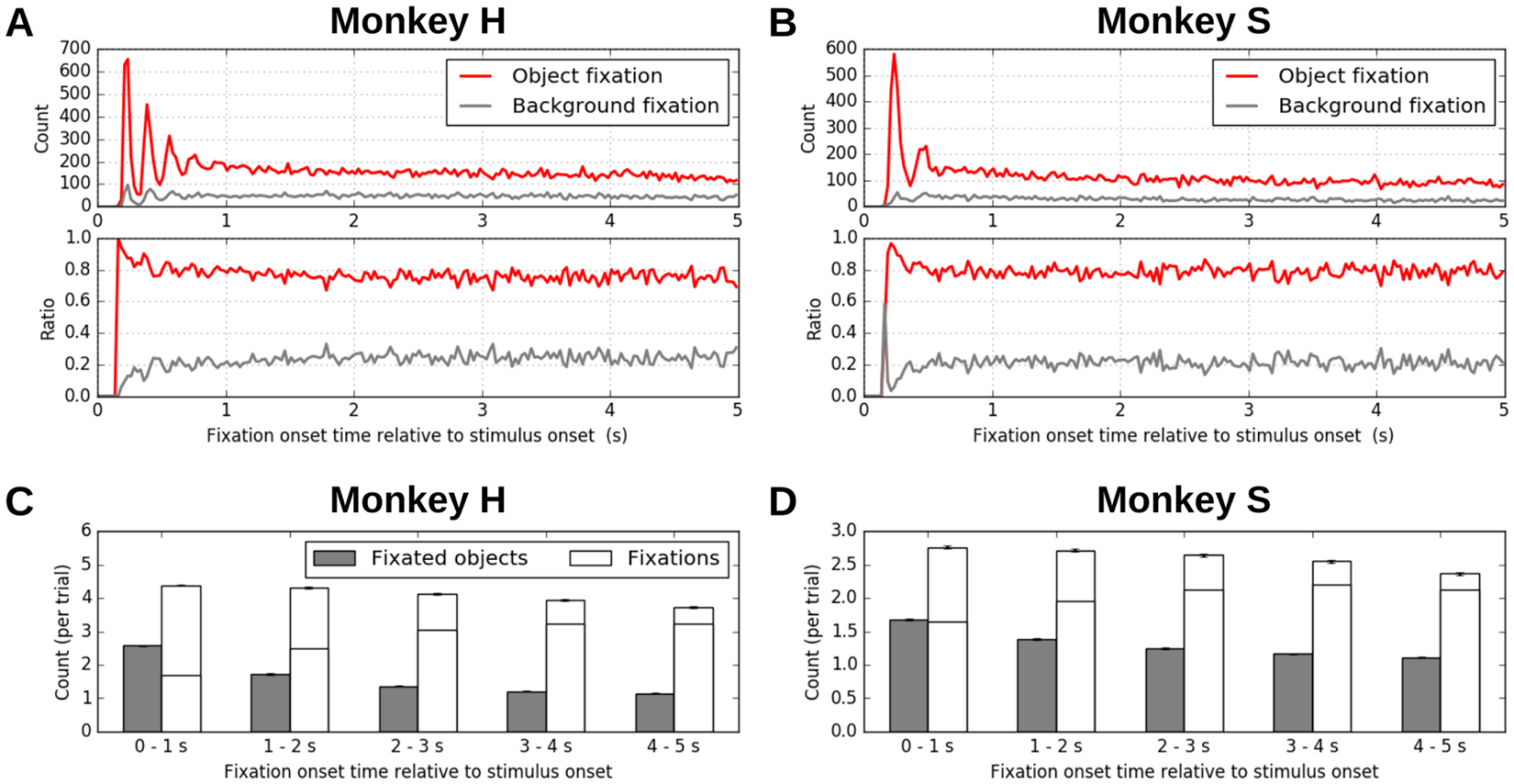
Temporal development of fixation behavior. (**A**) Top: distribution of fixation onset times computed separately for object fixation (red) and background fixation (gray) performed by monkey H. Bin width is 25 ms. Bottom: The ratio of the count in the top panel relative to the total count of fixations in each bin. The same color convention as for the top panel applies to the bottom panel. (**B**) Same plot as (**A**), but for monkey S. (**C**) The number of the fixated objects (gray) and the performed fixations (white) within each of 1-second intervals during free viewing trials of monkey H. The numbers were computed for 5 successive intervals covering the whole 5 second duration of the trials, as indicated below the bars. The bar heights represent the means over trials, and the error bars indicate the standard errors of the means. The tick in a white bar indicates the number of fixations per object, i.e., the number of the fixations divided by the number of the fixated objects in the corresponding interval. (**D**) Same plot as (**C**), but for monkey S.

Next, we were interested in the temporal evolution of the occurrence frequencies of the object fixations and the background fixations along the course of free viewing. Thus we computed, separately for the two types of fixations, histograms of fixation onset times (using 25 ms time bins) across the trials over the 5-second duration of a free viewing period (Fig. 3A and B, top). The histograms of both monkeys showed a few peaks in the initial period, reflecting that the latencies to the first few fixations after stimulus onset were highly consistent across trials. These peaks were followed by a slight, monotonic decrease in the fixation count toward the end of the trial. This general trend of decreasing fixation counts is a direct consequence of the increase in the fixation durations over time (Fig. 1C and D). The amounts of object fixations and background fixations normalized by the total number of fixations in each time bin (Fig. 3A and B, bottom) were almost constant over time, except for a higher amount of object fixation at the beginning of the trial. This initial behavior could be explained by the extremely high saliency of the objects by their sudden appearance at stimulus onset.

We further studied the temporal development of fixation behavior by counting, for each trial, how many objects were fixated within every 1-second interval, and by taking the average of the counts over trials (Fig. 3C and D, gray bars). Both monkeys showed a monotonic decrease in the number of fixated objects along the trial. It started with 2.57 for monkey H and 1.67 for monkey S in the first second of free viewing and converged to 1 in the last second for both monkeys. Note that this is not a consequence of the decrease in the number of fixations mentioned above. In fact, the number of fixations per object (Fig. 3C and D, ticks in white bars) increased over time, meaning that the monkeys made more fixations on a single object in later periods of free viewing than in the beginning. This result indicates that in the initial period of free viewing the monkeys performed an ambient inspection by exploring multiple objects and then shifted to focal examinations of single objects.

**Figure 4:**
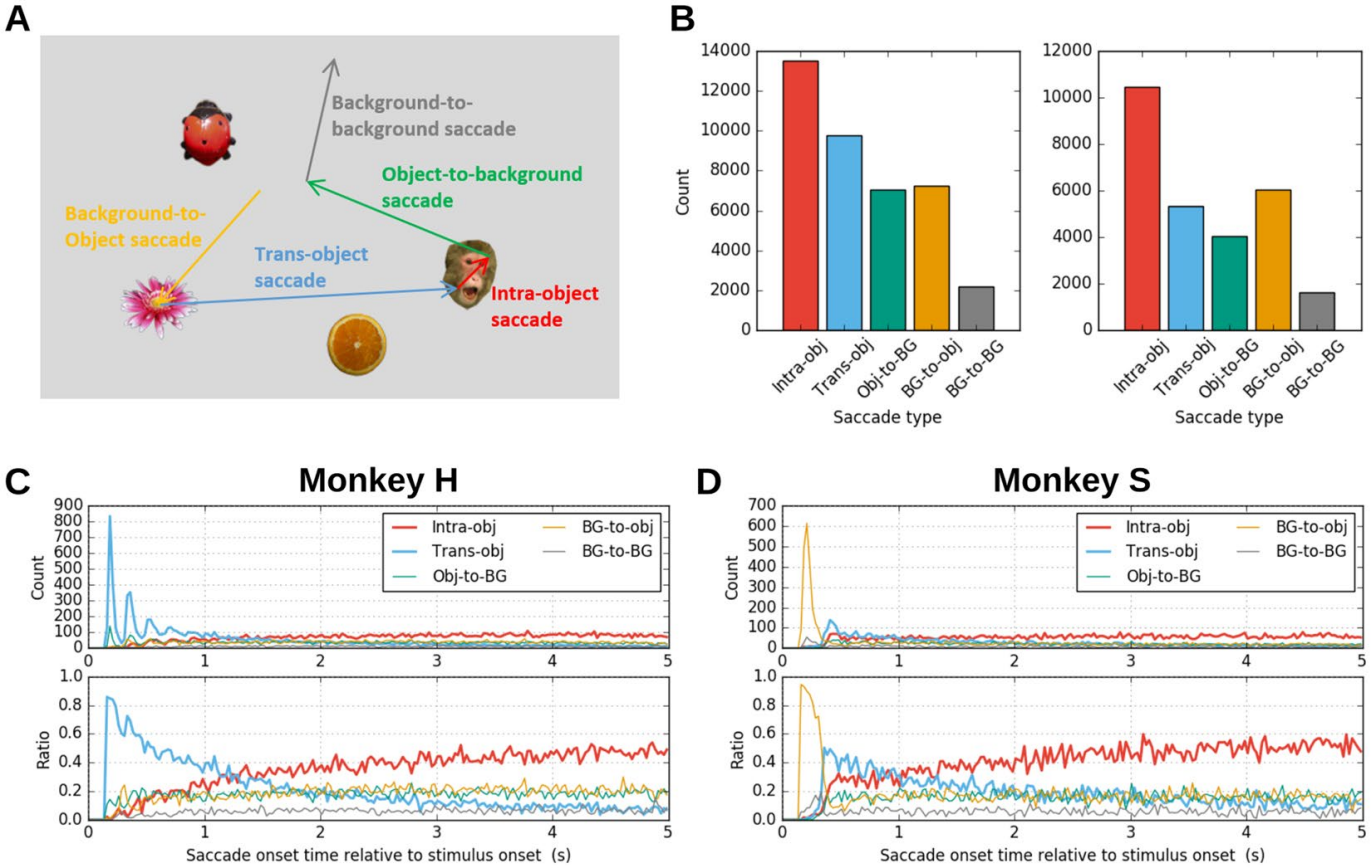
Temporal modulation of saccadic behavior. (**A**) Schematic illustration of saccade types. Object images are taken from the Microsoft image gallery. (**B**) The total number of saccades of each type. Colors correspond to them in (**A**). Left: ﻿monkey H. Right: monkey S. (**C**) Top: distribution of saccade onset times computed separately for saccades of each type (monkey H). Bin width is 25 ms. Bottom: the ratio of the counts of the top panel relative to the total number of saccades in each bin. Same color convention for both panels and as before. (**D**) Same plot as (**C**), but for monkey S. Note that the peak of the background-to-object saccade type in the beginning, which is missing in monkey H, is due to the difference in the stimulus images for the two monkeys (whether an object existed at the center of a stimulus image or not; see Methods for details).

Next, we were interested in the detailed sequence of the viewing behavior, in particular, if there were specific viewing patterns. Thus, we wanted to identify if a next fixation occurs on the same or another object, or on the background. Therefore we classified the saccades based on the positions of the fixations preceding and following each instance (Fig. 4A). This classification defined the following four saccade types: *object-to-object type* (preceded and followed by object fixations), *object-to-background type* (preceded by an object fixation and followed by a background fixation), *background-to-object type* (preceded by a background fixation and followed by an object fixation), and *background-to-background type* (preceded and followed by background fixations). The object-to-object type was further divided into *intra-object type*, representing saccades within an identical object, and *trans-object type*, representing saccades between two different objects. These latter two types constituted more than half of all saccades: 13,537 intra-object saccades (34.0%) and 9,790 trans-object saccades (24.6%) for monkey H, and 10,470 intra-object saccades (38.1%) and 5,333 trans-object saccades (19.4%) for monkey S (Fig. 4B). Note that the higher ratio of background-to-object saccades in monkey S than in monkey H was due to a slight difference in the stimulus images used for different monkeys, i.e., the stimulus images for monkey H always contained an object at the center, which was not the case for the stimuli for monkey S (for further details, see Stimulus Preparation in Methods).

The histograms of the saccade onset times for the different saccade types (Fig. 4C and D, top) showed that the trans-object saccades (in both monkeys) and the background-to-object saccades (in monkey S only) dominated the initial period (~1 sec) of free viewing, and then intra-object saccades took over the dominance in later periods. Note that the initial dominance of background-to-object saccades in monkey S, which is missing in monkey H, was again due to the difference in their stimuli (see Stimulus Preparation for further details). The time course of the ratio of each saccade type (Fig. 4C and D, bottom) illustrates this shift more clearly. At the beginning of free viewing trials, the ratio of intra-object saccades to all saccades was almost zero, but it increased to about 0.5 in the last second of the trials for both monkeys. Along with this increase, the ratio of the trans-object saccades, which was about 0.5 at around 500 ms after stimulus onset, kept decreasing until it reached below 0.1 for monkey H and 0.2 for monkey S at the end of the trial. This ratio indicates, in consistency with the observation on the number of fixated objects, that the monkeys performed in the initial period of the free viewing a rather global, cross-object exploration, and then shifted in a later period of the trial to a more focal examination of single objects.

**Figure 5:**
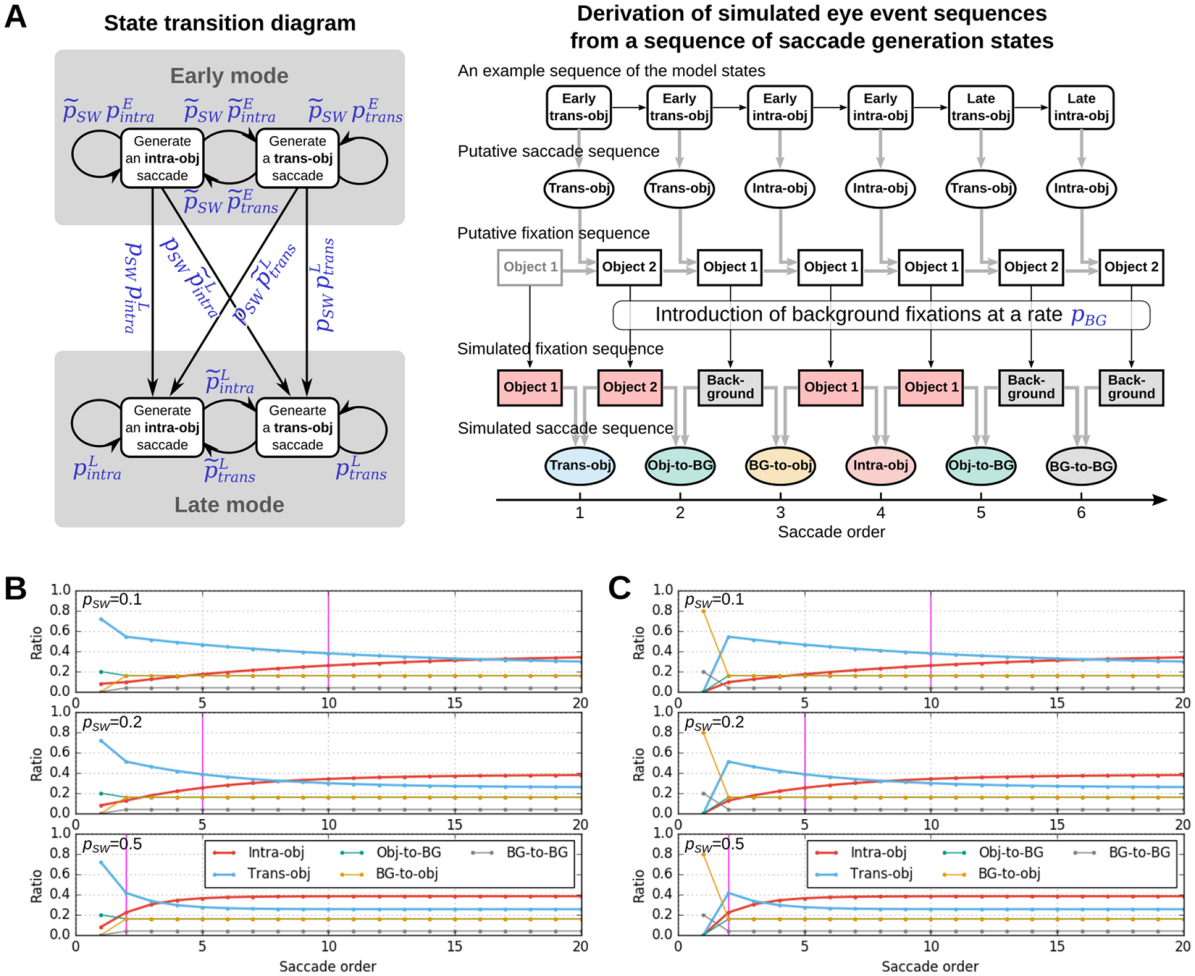
A stochastic model for the eye event sequence generation during free viewing. (**A**) Schematic illustration of the model. Left: Markov state transition diagram. Nodes represent the states of the model, and arrows indicate possible transitions between the states, with blue symbols next to them representing the associated transition probabilities. Note that symbols with tilde represent “one minus the symbol without tilde”, e.g., ${\tilde{p}}_{SW}=1-{p}_{SW}$. Right: Graphic representation of how the model generates simulated eye event sequences. Black and gray arrows indicate stochastic and deterministic dependences, respectively. See the main text and Methods for further details of the simulation procedure. (**B**) Example simulation results of saccade type ratios as functions of saccade order with different values of *p*_*SW*_ = 0.1, 0.2, 0.5, (from top to bottom, respectively). Other parameters were fixed arbitrarily at $({p}_{intra}^{E},{p}_{trans}^{E})=(0.1,0.9)$, $({p}_{intra}^{L},{p}_{trans}^{L})=(0.6,0.4)$, and *p*_*BG*_ = 0.2. To mimic the result of Monkey H, the first fixation was designated to be an object fixation irrespective of *p*_*BG*_. Magenta vertical lines indicate the expected mode-switch timing derived as the inverse of the corresponding *p*_*SW*_ values. (**C**) Same plots as in (**B**) except that the first fixation was designated to be a background fixation in order to replicate the behavioral paradigm of monkey S (no object placed in the center of the stimulus images).

In what follows we aim to model the observed shift in the visual exploration strategy. We base the model on the hypothesis of the existence of two visual processing modes that were reported in previous studies in humans^5, 11, 12^ postulating an ambient mode for initial global scanning and a focal mode for later local scanning. Thus we devised a generative model of saccade sequence that incorporates such two modes, i.e., an *early mode* and a *late mode*. The basic assumptions of the model are: (i) monkeys intend to fixate on an object at every saccade; (ii) however, due to limited accuracy of saccadic eye movements (see, e.g., ref. 13), monkeys erroneously make background fixations at a certain rate; (iii) when making a saccade, monkeys decide whether to fixate next on the currently fixated object (i.e., make an intra-object saccade) or on another object (i.e., make a trans-object saccade) in a stochastic manner; and (iv) the probabilities that govern this stochastic selection of saccade target depends on whether the monkey is in the early mode or the late mode of visual exploration; (v) the switch from the early mode to the late mode occurs only once at a random timing during a free viewing trial. To implement these assumptions, we formulated a discrete-time Markov state transition model (Fig. 5A). At each time step, the model generates a single saccade of a type determined by the current state of the model. According to assumptions (i) and (iv), the model consists of four saccade generation states (Fig. 5A, left): two pairs of an intra-object saccade generation state (or intra-state in short) and a trans-object saccade generation state (or trans-state in short), each of which pairs belongs to either the early mode or the late mode. The stochastic nature of the saccade target selection and the mode switch as stated in assumptions (iii) and (iv) is implemented as Markov transitions between these states governed by state transition probabilities as indicated in Fig. 5A (left). These state transition probabilities are defined in terms of the probability *p*_*SW*_ of the switch from the early mode to the late mode and the probabilities that govern state transitions within the early mode or the late mode: ${p}_{intra}^{E}$ and ${p}_{trans}^{E}$ are the probabilities to continue respectively the intra-state and the trans-state within the early mode, and ${p}_{intra}^{L}$ and ${p}_{trans}^{L}$ are the corresponding probabilities for the late mode. At the initial step the mode of the model is set to the early mode, and at each following time step the mode may switch to the late mode at the probability *p*_*SW*_. Once the mode switches to the late mode, it never switches back to the early mode, as stated in assumption (v). The erroneous occurrences of background fixations as stated in assumption (ii) are implemented by randomly flipping object fixations in a fixation sequence, which is simulated by the model as described below, to background fixations at a probability *p*_*BG*_. (See “Model description” in Methods for further details of model formulation).

To simulate a free viewing trial, the model first generates a sequence of saccade generation states (Fig. 5A right, “An example sequence of the model states”). These states generate a putative saccade sequence according to their type (Fig. 5A right, “Putative saccade sequence”). Given a saccade sequence, a consistent fixation sequence can be derived (Fig. 5A right, “Putative fixation sequence”). Here the number of objects is assumed to be two, and the first fixation is assumed to be on object 1, without a loss of generality. To this putative fixation sequence, background fixations are introduced by randomly flipping each fixation to a background fixation at the probability *p*_*BG*_. This fixation sequence after the introduction of background fixations constitutes a simulated fixation sequence. Finally, a simulated saccade sequence is derived from this fixation sequence in the same manner as for experimental saccade sequences. (See “Model description” in Method for further details of model simulation procedure).

We characterize the behavior of the model by the ratios of different saccade types as functions of saccade order. In order to simulate the ratios consistent to the empirically observed ones, first, *p*_*BG*_ is set to the empirically obtained ratio of the background fixations to all fixations (Fig. 2C and D), i.e., 0.23 for monkey H and 0.21 for monkey S. The remaining five parameters can be varied independently so that the saccade type ratios generated by the model become consistent with the empirical ratios. The ratios generated with different combinations of parameter values show different saccade order dependences. In particular, *p*_*SW*_ has a strong influence on the timing of the crossover between the ratios of trans-object saccades and intra-object saccades (Fig. 5B and C). Note that the inverse of *p*_*SW*_ represents the expected number of saccades before a switch from the early mode to the late mode (i.e., the expected number of Bernoulli trials until the first success, given *p*_*SW*_ as the success rate). For example, a mode-switch is most likely to occur at the 5th saccade when *p*_*SW*_ = 0.2.

**Figure 6:**
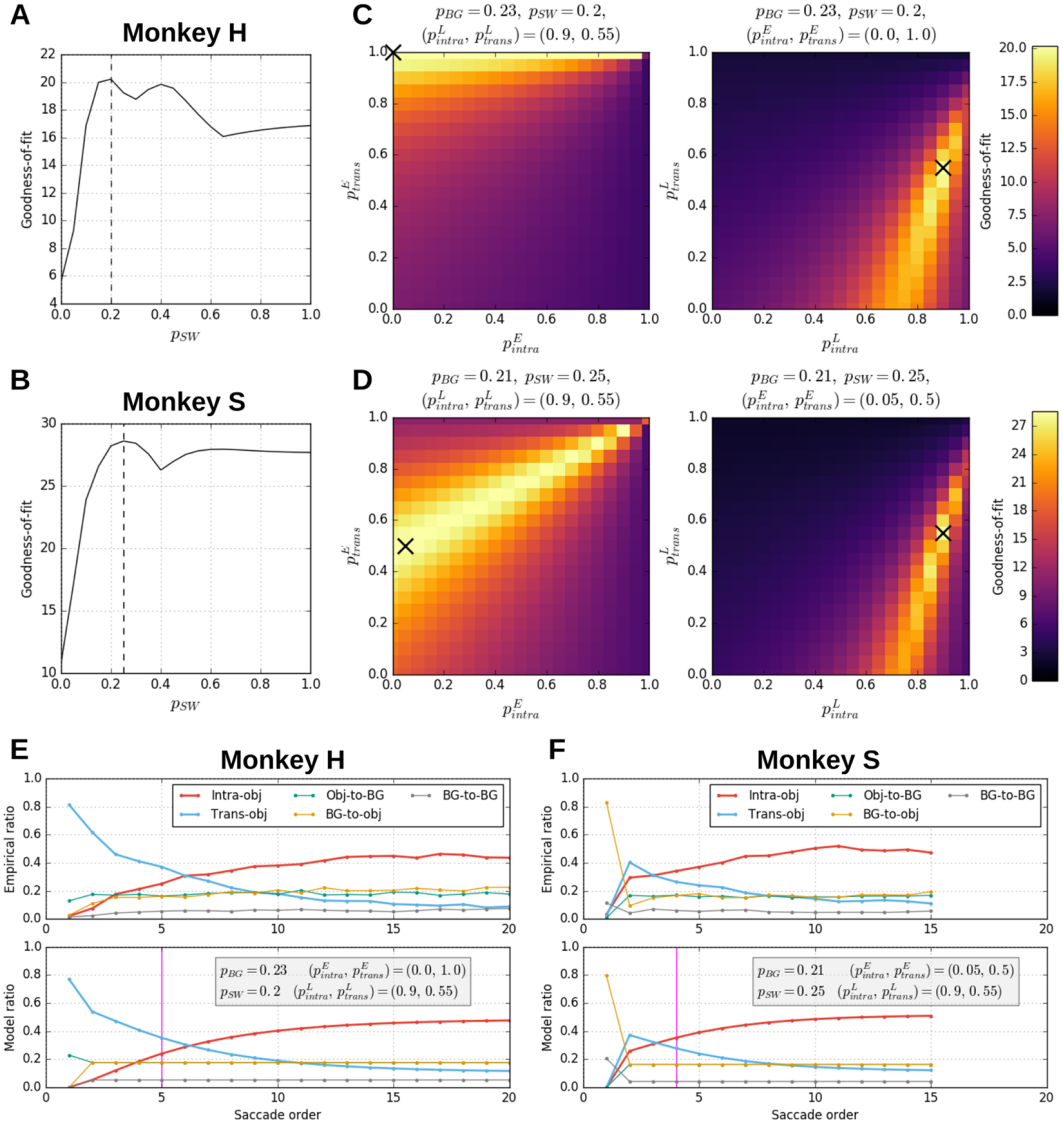
Dependence of the goodness-of-fit (GoF) measure on model parameters. (**A**) Dependence of the GoF measure on the switching probability *p*_*SW*_ obtained from the fitting of the model to the data of monkey H. The maximum GoF value achieved for each given *p*_*SW*_ value is plotted as a function of *p*_*SW*_. Note that the values of the other parameters than *p*_*SW*_ are varied for different *p*_*SW*_ values so that the GoF is maximized under the constraint of each given fixed *p*_*SW*_ value. The value of *p*_*SW*_ is changed in steps of 0.05. The (unconstrained) maximum GoF value is taken at *p*_*SW*_ = 0.20, as indicated by the vertical dashed line. (**B**) Same as (**A**), but for monkey S. The maximum GoF value is taken at *p*_*SW*_ = 0.25. (**C**) Dependence of the GoF measure on the transition probabilities in the early mode (${p}_{intra}^{E}$ and ${p}_{trans}^{E}$; left) and those in the late mode (${p}_{intra}^{L}$ and ${p}_{trans}^{L}$; right), obtained from the fitting of the model to the data of monkey H. The other parameter values (indicated above the panels) are fixed so that the maximum GoF value (indicated by the black crosses) is contained in the respective plots. GoF values are represented by colors as indicated by the color bar to the far-right. (**D**) Same as (**C**), but for monkey S. (**E**) Dependence of saccade type ratios on saccade order. Top: empirical ratios computed from the data of monkey H. Bottom: simulated ratios by the model with the best fitting parameter values (see Methods for how they are obtained), which are indicated in the gray box. The magenta vertical line in the bottom panel indicates the expected mode-switch timing derived as the inverse of *p*_*switch*_. (**F**) Same as (**E**), but for monkey S. Note that different initial conditions were used depending on the individual monkey to be consistent with the stimulus presentations in the experiment (monkey H: the initial fixation was on an object, monkey S: on the background).

The fit between the simulated and the empirical saccade type ratios is evaluated by a goodness-of-fit (GoF) measure which quantifies the similarity of the empirical and the simulated ratios, taking larger values for better fits (see Model description in Methods for definition). Using this measure we examined how the explanatory power of the model depends on the choice of the parameter values. To this end, we varied the values of *p*_*SW*_, ${p}_{intra}^{E}$, ${p}_{trans}^{E}$, ${p}_{intra}^{L}$ and ${p}_{trans}^{L}$ in steps of 0.05 and computed the GoF measure for every combination of the parameter values. This allows to plot the GoF as a function of the parameter values. We started with examining the dependence of the GoF on *p*_*SW*_ (Fig. 6A and B; note that the values of the other parameters than *p*_*SW*_ are varied for different *p*_*switch*_ values so that the GoF is maximized under the constraint of each given fixed *p*_*SW*_ value). The value of *p*_*SW*_ that gives the maximum GoF, which we call the best fitting value hereafter, was 0.2 for monkey H and 0.25 for monkey S. As the inverse of *p*_*SW*_ represents the expected number of saccades before a switch, our result indicates that a switch occurs at around fourth or fifth saccade on average. The GoF keeps relatively high values around the best fitting *p*_*SW*_ value, but it drops sharply at *p*_*SW*_ = 0. This means that a model without a switch has much less explanatory power than the one with a switch. This result may well be due to a lower number of effective parameters in the model without a switch (*p*_*SW*_ is fixed to 0, and ${p}_{intra}^{E}$ and ${p}_{trans}^{E}$ have no effect) than in the full model. To take this into account, we compared the two models in the framework of model selection based on the Akaike’s information criterion (AIC), which is a measure of the relative quality of statistical models for a given set of data. For both monkeys, the model with a switch has a smaller AIC value than the model without a switch: AIC values of the models with and without a switch are 9.88 · 10^5^ and 1.03 · 10^6^ for monkey H and 6.84 · 10^5^ and 6.99 · 10^5^ for monkey S, respectively, meaning that the model with a switch gives a better prediction of the given data than the model without a switch even after penalization for more effective parameters in the former model than the latter.

We proceed to examine the dependence of the GoF on the state transition probabilities. We start with the dependence on the state transition probabilities in the early mode, i.e., ${p}_{intra}^{E}$ and ${p}_{trans}^{E}$. To do that, we fix the other parameters at their best fitting values and study how the GoF varies in the $({p}_{intra}^{E},{p}_{trans}^{E})$-plane (Fig. 6C and D, left). The maximum GoF value is at $({p}_{intra}^{E},{p}_{trans}^{E})=(0.00,1.00)$ for monkey H and (0.05, 0.50) for monkey S. For both monkeys the best fitting value of ${p}_{trans}^{E}$ is much higher than that of ${p}_{intra}^{E}$, indicating that the generation of trans-object saccades tends to continue in the early mode much more likely than the generation of intra-object saccades. This is consistent with the interpretation of the ambient exploration in the early phase of free viewing. The GoF values get lower as ${p}_{intra}^{E}$ gets higher and ${p}_{trans}^{E}$ gets lower than their best fitting values, meaning that the explanatory power of the model becomes weaker as the early mode loses the character of ambient exploration.

We then study the dependence of the GoF on the state transition probabilities in the late mode, i.e., ${p}_{intra}^{L}$ and ${p}_{trans}^{L}$, in the same manner as for the early mode (Fig. 6C and D, right). The maximum GoF is at $({p}_{intra}^{L},{p}_{trans}^{L})=(0.90,0.55)$ for both monkeys. Thus, in contrast to the early mode, the best fitting value of ${p}_{intra}^{L}$ is higher than ${p}_{trans}^{L}$ for both monkeys, which is consistent with the interpretation of the focal exploration in the late period of free viewing. In the $({p}_{intra}^{L},{p}_{trans}^{L})$-plane, high GoF values are found only below the diagonal line. This means that the explanatory power of the model becomes weak as the late mode loses the character of focal exploration.

Comparison between the empirical saccade type ratios and the ones generated by the model with the best fitting parameter values (Fig. 6E and F) illustrates that the model reproduces well the saccade order dependence of the saccade type ratios for both monkeys in various aspects: (a) the monotonic increase and decrease of the ratios of intra-object saccade and trans-object saccade, respectively, (b) the timing of the cross-over of these ratios, (c) the constancy of the ratios of object-to-background, background-to-object, and background-to-background saccades, and (d) the overall quantitative values of all the ratios. These features can also be reproduced when the model is applied to the data from the trials with natural scene background stimuli (“Application of the model to free viewing eye movements on natural scene background stimuli” in Supplementary Information), but with a reduced performance (in terms of GoF value) due to increased background fixations induced by the complex background images. Altogether, these results suggest that our model is flexible and powerful enough to explain dynamical features of free viewing eye movements on different types of complex images.

## Discussion

In this work, we aimed to elucidate the relationship of eye movements in monkeys to the ambient and the focal processing modes. We demonstrated that these processing modes correlate with saccades across and within visual objects, respectively. We also showed, by employing a novel modeling approach, that the change from an ambient mode to a subsequent focal mode could occur as an abrupt switch in the exploration mode during free viewing.

There is still a controversy on whether visual saliency^14, 15^ or visual objects^16–19^ better account for visual fixations on natural scenes. Independently of the putative weight of these factors to explain fixation positions, we found that there is a time-dependence of the focus of visual attention, with a decrease in the number of object fixations as the trial progressed, accompanied by an increment in the successive fixations within the same visual objects. The visual behavior of the monkeys in our study agrees with aspects of other reports on human viewing behavior^5–9^. These studies analyzed eye movement behavior in humans during free viewing and related the behavior to fixation duration and saccade amplitude. They observed during the time course of free viewing a decrease in fixation duration and a concurrent increase in saccade amplitude, consistent with what we reported here in monkeys. The common interpretation was that early on during the free viewing a global scanning (ambient) mode is dominant and is later replaced by a dominance of local exploration. Such eye movement behavior emerges without any specific training or a specific behavioral request such as those in a visual search or memory task.

Based on the common findings, we aimed to characterize these viewing modes by additionally considering the type of saccades during free viewing. We asked the following questions: (a) would these two viewing modes imply that there is a characteristic set of probabilities of different saccade types, such as trans-object and intra-object saccades? And if that is the case, (b) could this change in exploration mode occur as an abrupt switch instead of a gradual shift as it was assumed from the dynamics of saccade and fixation? We were able to answer these two questions based on an in-depth statistical analysis of the dynamics of the different type of saccades, dependent on where the previous and following fixations were, and by modeling the eye movements by a Markov state transition model. Our results directly demonstrate that the global scanning is concerned with scanning many objects spread in the scene and the local scanning concentrates on viewing single objects. This behavior can be quantitatively reproduced by our model for the generation of eye movement sequences with an abrupt switch between the ambient and the focal saccade generation modes. These results have three direct implications. First, our findings provide a solid support for the two-mode hypothesis, which postulates distinct ambient and focal processing modes. Also, our results demonstrate the possibility of abrupt switches from ambient to focal modes and, finally, that the dynamics of these modes seem to be shared between humans and monkeys.

While our results are in favor of an abrupt switch between the two processing modes, they do not exclude the possibility that a model with a gradual shift from the ambient to the focal mode might give an equally good or even better explanation of given data. Comparing the two types of models, a notable feature of the former is that it can be used to infer the most probable timing of a switch from eye movement data of a single free viewing trial. If such a model captures the fundamental mechanisms underlying the generation of saccade sequences during free viewing, we would expect that the brain exhibits a certain abrupt change in its activity coincident with the inferred switch timing. Such a change would not be observed if the mode shifts gradually. Thus, analysis of neuronal activities during free viewing would provide clues to judge whether an abrupt switch or a gradual shift is more plausible to explain the eye movement dynamics. Previous studies have suggested possible relations between neuronal activity and the distinct visual processing modes. Trevarthen^20^ hypothesized that the ambient and focal modes correspond to the engagement of different systems, i.e. subcortical and cortical visual systems, respectively. Alternatively, Velichkovski *et al*.^11^ and Unema *et al*.^5^ proposed a particular role of the dorsal and ventral cortical visual streams in those respects. Furthermore, previous studies on neuronal activities in the primary visual cortex of freely viewing capuchin monkeys^21–23^ and the hippocampus of visually searching macaque monkeys^24^ reported intimate relationships between oscillatory activities in these areas and the saccade rhythm. Elucidating such relationships in our recordings of neuronal activities and detecting their changes related to the ambient and focal processing modes will be a crucial test of the two-mode hypothesis and our model of saccade sequence generation.

Some previous studies reported analytical approaches aimed to explain eye movement behavior. Most models concentrate on explaining which locations of a given image (or a frame of a movie) are likely to be fixated, and are based on the saliency computed from the image^3^. This type of modeling typically focuses on deriving a static saliency map that predicts gaze-attracting regions in a given image, while mostly ignoring the temporal evolution of saccade target selection (for exceptions see refs 25–30). Our model is complementary to the existing models based on visual saliency while introducing dynamical aspects to eye movement modeling. The model enables to infer the timing of the switch between the two modes. How this switching timing varies depending on task conditions other than free viewing are interesting open questions for future research.

In our model background fixations were introduced as random errors at a constant rate, based on the constant ratio of background fixations observed in the empirical eye movement data (Fig. 3A and B). A likely cause of such errors is systematic overshoot or undershoot of saccadic endpoints^31, 32^. Previous studies have reported that the displacement of a saccade endpoint from the saccade target grows proportionally to the saccade amplitude^31, 33^. This suggests that the decrease in saccade amplitude over the course of free viewing (Fig. 1D) should also lead to a decrease in the ratio of background fixations. This is, however, contradictory to the observation of a constant background fixation ratio in our data (Fig. 3A and B). This implies a modulation of saccade accuracy over the course of free viewing. While in the present modeling attempt we took a phenomenological approach to introduce background fixations at a constant rate derived from the empirical data, one could think of a more realistic model based on a mechanism of the saccade overshoot and undershoot^34–36^. Such a model needs, however, to also incorporate a mechanism for modulation of saccade accuracy, in order to account for the constant background fixation ratio. Formulation and validation of such a mechanism would require further dedicated experiments.

Apart from the interest in intra-cranial neuronal recordings, studying monkey eye movements per se has its own relevance^37–40^. Particularly, our data may help to reconcile the controversy between visual saliency or visual object as the dominant factor in saccade target selection^15^, because monkeys may lack any instructional bias apparent in human studies. In a wider perspective, while visual exploration with saccadic gaze shifts is observed in a large range of species, beyond primates or mammals, including birds, fishes, and insects^41^, the commonality in visual exploration strategy across species has never been studied systematically. Overall, our analysis of monkey eye movements provides evidence that the notion of ambient and focal processing modes characterized in humans can be generalized to the free viewing behavior of monkeys. The experimental paradigm and the analysis methods developed in the present study can be directly employed to data from other experiments.

## Methods

### Experiment

#### Animal preparation

We implanted in two macaque monkeys (*Macaca fuscata*), monkeys H (body weight 7.1 kg, female) and S (body weight 5.2 kg, female), a head restraint. The rest of the exposed part of the skull was covered with acrylic resin. This surgery was performed under full anesthesia by inhalation of 1–3% isoflurane (Forane, Abbott Japan, Japan) in nitrous oxide (70% N_2_O, 30% O_2_) through an intratracheal cannula. An antibiotic (Pentcilin, Toyama Chemical, Japan; 40 mg/kg, im) and an anti-inflammatory and analgesic agent (Voltaren, Novartis, Tokyo, Japan; 12.5 mg, or Ketoprofen, Nissin Pharmaceutical, Yamagata, Japan; 0.6 mg/kg im) were given immediately after the surgery and continued during the first postoperative week. After 1–2 weeks of recovery, scleral search coils for the measurement of the eye position were implanted in the left eye under the same anesthesia as mentioned above. All experiments were performed in accordance with the guidelines of the National Institute of Health (1996) and the Japan Neuroscience Society, and were approved by the Osaka University Animal Experiment Committee (certification No. FBS-13-003).

#### Behavioral Task

The monkeys performed a free viewing task. They sat in a primate chair with their heads fixed. An LCD monitor (FlexScan EV2736W-FS, EIZO, Japan) for stimulus presentation was placed in front of the chair at a distance of 57 cm from the eyes of the animals. A trial (Fig. 1A) started with a presentation of a fixation spot (a white or colored square of 0.2 deg edge lengths on a gray background) at the center of the monitor screen. As soon as the animals maintained a fixation on the fixation spot for 500 ms, the spot was turned off and a stimulus image for free viewing was presented. After the onset of a stimulus image, the animals were free to move their eyes except shifting their gaze outside the boundaries of the image. The image was turned off five seconds after the onset of its presentation and the monkeys were rewarded with a drop of juice or water. If the animals’ gaze went beyond the image boundaries before the end of the five seconds, the trial was immediately aborted and no reward was given. The interval between the reward and the appearance of the fixation spot initiating the next trial was 2.0 sec (for monkey H) or 1.5 sec (for monkey S). The experiment continued as long as the animals’ motivation lasted (typically more than 30 min).

#### Stimulus preparation

The stimulus images for the free viewing experiment were generated by placing 5 object images on a background image. 5 objects were randomly selected from a set of 64 object images (see Supplementary Fig. S1) of 2 deg in diameter on average and were placed within a 34.8 × 26.1 deg^2^ background image, which was either a solid gray background or one of 67 natural scene images (see Fig. 1B for example stimulus images). The object images were taken by one of the coauthors or drawn from Microsoft image gallery (used to be available on Office Online: https://www.office.com), used with permission from Microsoft. The background images were taken by one of the coauthors or drawn from the Natural Image Database of Center for Perceptual Systems, University of Texas at Austin (http://natural-scenes.cps.utexas.edu/db.shtml; ref. 42) and the McGill calibrated color image database (http://tabby.vision.mcgill.ca/; ref. 43). We created slightly different sets of stimulus images for the purpose of electrophysiological data collection done in parallel to behavioral data collection. For the analysis in the present study, we used one of such stimulus sets. The objects were placed on the background using the following procedure: for monkey H we first placed one object at the center of the image, and then placed the other objects one by one at random positions avoiding previously placed objects so that the centers of any two objects were separated by at least 4 deg. The same procedure was used for monkey S except that the first object was not restricted to the center of the background but placed at an arbitrary position. Due to this construction, the first saccade of monkey H after stimulus onset always started from the object placed at the center of the stimulus image, where the monkey had been fixating before stimulus onset. On the contrary, the first saccade of monkey S most likely started from the background at the center of the screen, where the monkey also fixated before stimulus onset. This difference resulted in the differences in the measures shown in Fig. 4B,C and D.

#### Eye tracking

The eye positions during the free viewing task were recorded with a scleral search coil system (DSC2000, SANKEI, Japan) at a sampling rate of 20 kHz and stored on hard disk drives for offline analysis. We collected these data from monkey H from 1,983 gray background trials and 3,126 natural scene background trials over a total of 25 recording sessions (one or two sessions per day), and data of monkey S from 2,609 gray background trials and 4,863 natural scene background trials over 79 recording sessions (one or two sessions per day).

### Analysis

#### Eye event detection

We extracted the periods of saccades and fixations from the recorded eye position data by the following procedure. First, we estimated the velocity and the acceleration of the eye movements by computing the temporal derivatives of the data using the respective Savitzky-Golay filters (filter size: 199 samples, corresponding to 10 ms; polynomial order: 2)^44, 45^. Then, we collected the time segments throughout which the eye velocity and the acceleration were above 30 deg/s and 8,000 deg/s^2^, respectively, as potential saccade periods. The segments with either a peak velocity exceeding 1,500 deg/s, or a peak acceleration exceeding 120,000 deg/s^2^, or a duration shorter than 5 ms, or a duration longer than 100 ms, or a gaze shift shorter than 0.1 deg were considered as artifacts or noise, and were discarded. We made no discrimination between microsaccades and normal saccades. The remaining segments were identified as saccade periods, and the start and end times of each segment were stored as saccade onset and offset, respectively. Finally, periods between two successive saccade periods were identified as fixation periods unless the gaze shifts exceeded 1 deg. Saccades and fixations initiated between 0 and 150 ms from stimulus onset were discarded from the analysis, because they are likely not related to the processing of the presented stimulus, considering a minimum saccadic response latency of macaque monkeys of about 150 ms (see, e.g., ref. 46).

#### Eye event parameters

We analyzed the statistics of eye movements in terms of the following eye event parameters: the *saccade amplitude*, i.e., the distance between the eye positions at the onset and at the offset of a saccade; the *fixation position*, i.e., the average eye position between the offset of the preceding saccade and the onset of the following saccade; the *fixation duration*, i.e., the time interval between the offset of the preceding saccade and the onset of the following saccade.

To quantify the distribution of the fixation positions in relation to the objects in the presented stimulus image, we defined an additional fixation parameter, the *object distance*, as the distance between the fixation position and the center of an object. The object distance was computed between each fixation and each of the objects in the viewed stimulus image, yielding 5 object distances per fixation. The shortest of the 5 object distances of a given fixation, termed *shortest object distance*, indicates how close that fixation was to either of the 5 objects in the presented stimulus image. The distribution of shortest object distances across fixations characterizes the distribution of fixation positions in relation to the objects in the stimulus images (Fig. 2C and D).

#### Classification of fixations and saccades

We classified fixations, based on their shortest object distances, into the ones made on objects (*object fixations*), and the ones on the background (*background fixations*) according to the following criterion. Fixations of which shortest object distances were less than a predefined threshold value of 1.5 deg were defined as object fixations, and all the other fixations as background fixations. We chose the threshold value of 1.5 deg considering the extent of the object images (~1 deg in radius) and the size of foveal vision (<1 deg in radius). However, we also confirmed that the results do not depend on a specific choice of this threshold if chosen in the range between 1 to 2 deg. For each object fixation, we defined the *fixated object* as the object closest to the fixation position (in other words, the object that gave rise to the shortest object distance of the fixation). For background fixations their fixated object was not defined.

Saccades were classified, based on the types of their preceding and following fixations, into the following five types: *intra-object saccade*, of which the preceding and following fixations were on an identical object; *trans-object saccade*, of which the preceding and following fixations were on different objects; *background-to-object saccade*, which was preceded by a background fixation and followed by an object fixation; *object-to-background saccade*, which was preceded by an object fixation and followed by a background fixation; and *background-to-background saccade*, which was preceded and followed by background fixations.

### Modeling

#### Model description

To explain the observed temporal evolution of the ratios of different saccade types relative to the total number of saccades (Fig. 4C and D) we devised a discrete-time Markov state transition model for the generation of saccade type sequences. A mathematically concise representation of the model is given in Supplementary Information (“Mathematical formulation of the Model”). Here we provide additional details of the model based on the model description given in the Results section.

The model has four saccade generation states, i.e., two pairs of the *intra-object saccade generation state* (or *intra-state* in short) and the *trans-object saccade generation state* (or *trans-state* in short), each of which pairs belongs to either of two modes, i.e., *early mode* and *late mode* (Fig. 5A, “State transition diagram”). This model structure, composed of the states engaged in the generation of different types of saccade, is motivated by the results of a supplementary analysis of the eye movement data (“Sequential dependence between successive saccade types” in Supplementary Information). This analysis reveals repetitions of intra-object and trans-object saccades occurring significantly more frequently than expected by chance, suggesting a need for a model to incorporate a mechanism to generate such repetitions. We implement such a mechanism by constructing the model to possess saccade generation states for each of the two types of saccades, and parameterizing the degree of repetitive generation of a single saccade type with the probabilities of transitions between the states as follows.

The model changes its state at each simulation time step according to the state transition probability associated with each possible transition between states (blue symbols in Fig. 5A, “State transition diagram). The transition probabilities are factorized by the following five unitary probabilities: *p*_*SW*_, ${p}_{intra}^{E}$, ${p}_{trans}^{E}$, ${p}_{intra}^{L}$ and ${p}_{trans}^{L}$. The first one represents the probability of the switch from the early mode to the late mode (more precisely, the transitions from one state in the early mode to another state in the late mode), and the latter four concern the transitions between two states within a mode. Specifically, in the late mode, a transition from the intra-state to the trans-state occurs at a probability $1-{p}_{intra}^{L}$ (in other words, the model takes the intra-state repeatedly at a probability ${p}_{intra}^{L}$), and a transition from the trans-state to the intra-state occurs at a probability $1-{p}_{trans}^{L}$ (or the model takes the trans-state repeatedly at a probability ${p}_{trans}^{L}$). The same applies to the early mode, but, since the mode can switch to the late mode at a probability *p*_*SW*_, the respective transition probabilities are multiplied by 1−*p*_*SW*_, i.e., the probability for the switch not to occur.

A model simulation is initiated by setting the initial state of the model to either the intra- or the trans-state of the early mode. These states are taken obeying the probabilities $(1-{p}_{trans}^{E})/(2-{p}_{intra}^{E}-{p}_{trans}^{E})$ for the intra-state and $(1-{p}_{intra}^{E})/(2-{p}_{intra}^{E}-{p}_{trans}^{E})$ for the trans-state, which are the asymptotic occurrence probabilities of the intra- and trans-state, respectively, in an infinitely long Markov chain obeying the state transition rule of the early mode. The model states at the succeeding time steps are generated obeying the state transition probabilities (Fig. 5A, “An example sequence of the model states”). According to the state at each step, a saccade of the corresponding type is generated (Fig. 5A, “Putative saccade sequence”). The generated sequence of saccade types is then used to derive a corresponding fixation sequence. We assume, for simplicity without losing generality, that there are only two objects (object 1 and object 2) in the stimulus image. The initial fixation is set on object 1 (for simulation of monkey H) or the background (for simulation of monkey S), according to the different stimulus preparation for the different monkeys (see Stimulus Preparation in Methods). The subsequent fixations are generated such that they are consistent with the saccade type sequence (Fig. 5A, “Putative fixation sequence”).

Up to this step no background fixations are generated (except for the initial fixation for monkey S) since the model generates only intra-object saccades or trans-object saccades. They are introduced by converting a portion of fixations, which are randomly chosen from the derived fixation sequence at a probability *p*_*BG*_, to background fixations. This converted fixation sequence is the outcome of the model representing a simulated fixation sequence (Fig. 5A, “Simulated fixation sequence”). The corresponding saccade sequence is derived in the same manner as we classify empirical saccades based on preceding and following fixations (Fig. 5A, “Simulated saccade sequence”). The length of the fixation sequence for a simulation of one trial is determined according to the average number of fixations in one trial, i.e., 21 for monkey H and 16 for monkey S.

#### Model fitting

The model possesses 6 parameters: the probability *p*_*BG*_ of generating a background fixation, the probability *p*_*SW*_ of switching from the early to late mode, the probabilities $({p}_{intra}^{E},{p}_{trans}^{E})$ of staying in the current state in the early mode, and the corresponding probabilities $({p}_{intra}^{L},{p}_{trans}^{L})$ in the late mode. These parameters were adjusted so that the time courses of the fixation and saccade type ratios derived from simulations fitted to the empirical time-dependent ratios. First, to fit the fixation type ratios, ${p}_{BG}$ was set to the empirical proportion of background fixations to all fixations, which was 0.23 for monkey H and 0.21 for monkey S. Then, to fit the saccade type ratios, the remaining 5 parameters were independently varied in steps of 0.05 and a goodness-of-fit (GoF) measure defined as $GoF={(\frac{1}{{N}_{fix}-1}\sum _{i=1}^{{N}_{fix}-1}|{r}_{i}^{{\rm{emp}}}-{r}_{i}^{{\rm{sim}}}|)}^{-1}$, where ${r}_{i}^{{\rm{emp}}}$ is a 5-dimensional vector representing the empirical ratios of the 5 saccade types at the *i*-th saccade after trial start, ${r}_{i}^{{\rm{sim}}}$ is the corresponding ratio of the simulated data, and *N*_*fix*_ is the length of the simulated fixation sequence per trial (see Model description in Methods). The GoF measure was computed for each and every combination of the 5 parameter values. The best-fitting combination was identified as the one with the maximum GoF measure over all combinations, i.e., $({p}_{switch},{p}_{intra}^{E},{p}_{trans}^{E},{p}_{intra}^{L},{p}_{trans}^{L})=(0.20,0.00,1.00,0.90,0.55)$ for monkey H and $({p}_{switch},{p}_{intra}^{E},{p}_{trans}^{E},{p}_{intra}^{L},{p}_{trans}^{L})=(0.25,0.05,0.50,0.90,0.55)$ for monkey S.

## Electronic supplementary material

Supplementary Figures and Text
