# Supplementary material for: Switch from ambient to focal processing mode explains the dynamics of free viewing eye movements
Source: Sci Rep. 2017 Apr 24;7:1082. doi: 10.1038/s41598-017-01076-w (PMC5430715; doi:10.1038/s41598-017-01076-w)
Supplement: Supplementary file 1 — Supplementary Figures and Text [file 41598_2017_1076_MOESM1_ESM.pdf]

Supplementary Information for

SWITCH FROM AMBIENT TO FOCAL PROCESSING MODE EXPLAINS THE DYNAMICS OF FREE VIEWING EYE MOVEMENTS

Junji Ito[1*†], Yukako Yamane[2,3†], Mika Suzuki[2], Pedro Maldonado[4], Ichiro Fujita[2,3], Hiroshi Tamura[2,3], Sonja Grün[1,2,5]

[1]Institute of Neuroscience and Medicine (INM-6) and Institute for Advanced Simulation (IAS-6) and JARA BRAIN Institute I, Jülich Research Centre, Jülich, Germany. [2]Graduate School of Frontier Biosciences, Osaka University, Osaka, Japan. [3]Center for Information and Neural Networks, Osaka, Japan. [4]BNI, CENEM and Programa de Fisiología y Biofísica, ICBM, Facultad de Medicina, Universidad de Chile, Santiago, Chile. [5]Theoretical Systems Neurobiology, RWTH Aachen University, Aachen, Germany.

([*]Correspondence to j.ito@fz-juelich.de)

([†]These authors contributed equally.)

Object images used in the experiment

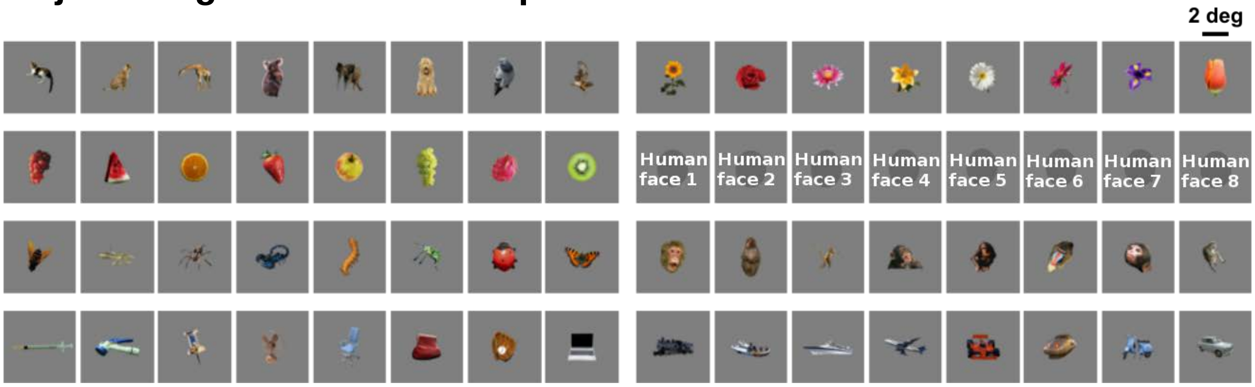

Supplementary Figure 1. The whole set of 64 object images used to generate stimulus images for the free viewing task. The scale bar to the upper right indicates 2 degrees of visual angle. “Human face” 1-8 are facial images of different individuals, not shown here for anonymisation reasons. Gray disk in these images indicates the size of the faces. All images are drawn from the Microsoft image gallery (see Methods of the main article for details), except for the first two human face images, which were taken by one of coauthors.

Sequential dependence between successive saccade types

In this analysis we examine the temporal structure of free viewing eye movements on a finer temporal scale than the analysis in the main article concerns about, by focusing on sequential dependences between types of successive saccades. To this end, we formulate a framework to test the significance of successive occurrence of saccade types for every possible combination of the types. For simplification, we focus on pairs of saccades which immediately precede and follow an object fixation (Fig. S2A). We test whether a specific combination of saccade types occurs more or less frequently than expected by chance. Thus, the null hypothesis for this test is defined as: the types of saccades preceding and following an object fixation

are independent of each other. Rejection of this null hypothesis means that there is a certain bias in the occurrence pattern of successive saccade types.

For each possible combination of saccade types, we counted occurrences of successive saccade pairs of which types match the combination in the empirical eye movement data of the monkeys. From the empirical count for all combinations, we constructed a 3x3 contingency table, of which the rows are different types of the preceding saccades (i.e., intra-object, trans-object and background-to-object) and the columns are different types of the following saccades (i.e., intra-object, trans-object and object-to-background), by filling the cells with the corresponding counts. We applied a χ^2-test to the table to test for deviation of the empirical counts from the counts expected under the null hypothesis of independence. In case of significant deviation, we further applied a post-hoc residual analysis to each of the empirical counts in order to separately test the significance of the deviation of each empirical count from its expected count.

The results of this analysis are summarized in a form of transition diagram (Fig. S2B and C). Both monkeys show significantly more frequent successive intra- and trans-object saccades (red circular arrows at the intra-object node and the trans-object node), while the transitions between these types occur significantly less than expected by chance (blue arrows in both directions between the intra-object node and the trans-object node). Thus, the analysis reveals that saccades of a same type tend to occur in successive repetitions. This result motivated us to construct the saccade sequence generation model, described in the main article, to be composed of distinct states engaged in generation of specific types of saccade, in order to incorporate a mechanism for generation of the successive repetitions revealed in the empirical data.

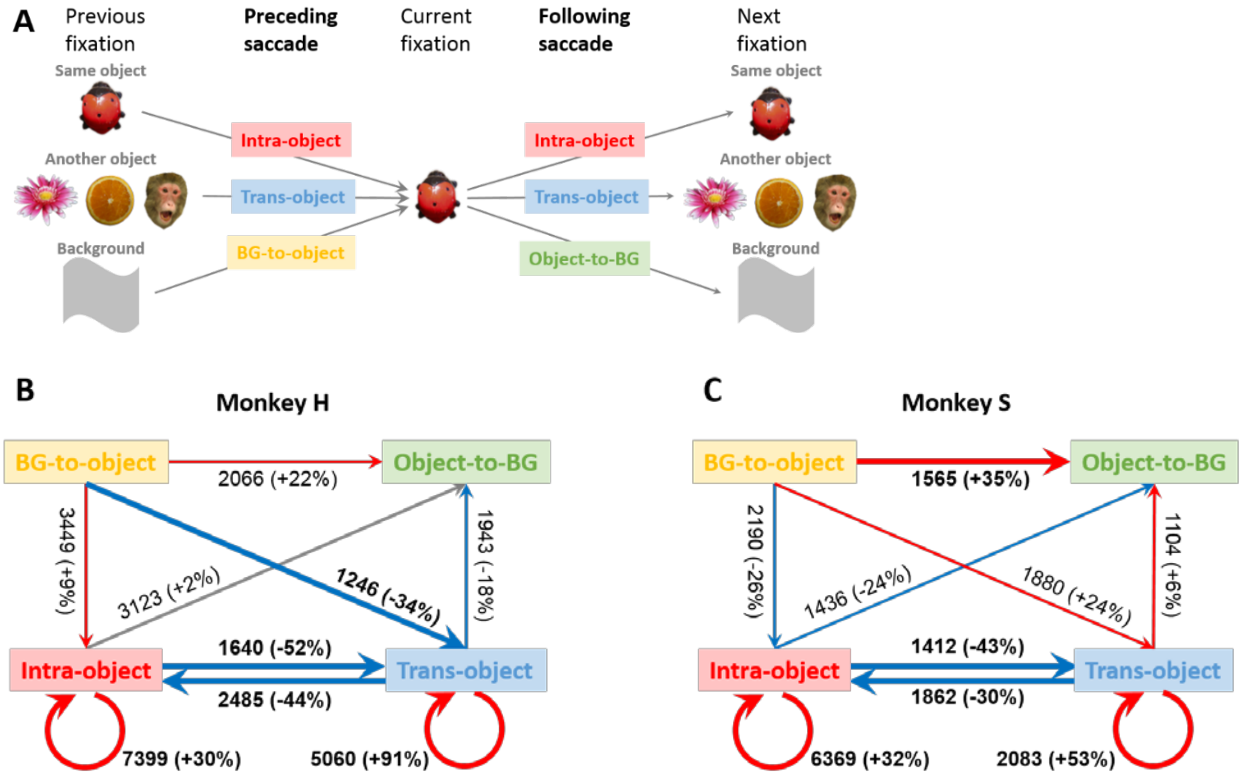

Supplementary Figure 2. Sequential dependence between successive saccade types. (A) Schematic illustration of the saccade types that we focus on in the present analysis. We test for biases in the combination of saccade types preceding and following an object fixation. Object images are taken from the Microsoft image gallery. (B) Graphical summary of the test performed on the whole saccade data of monkey H. Nodes and arrows represent saccade types and their transitions, respectively. Circular arrow represents

recurrence of an identical saccade type. Numbers next to the arrows indicate the empirical occurrence count of the respective transitions and recurrences, with the percentage values in parenthesis representing the difference of the empirical count from the occurrence counts expected by chance (e.g., “+50%” means that the empirical count is 150% of the expected count). Thick arrows indicate the transitions and recurrences with the difference percentages more than 30% or less than -30%. Color of the arrows indicates significantly more (red), less (blue), or non-significant (gray) empirical count than the expected. Significance level was set to p = 0.01. (C) Same graph as (B), but for monkey S.

Application of the model to free viewing eye movements on natural scene background stimuli

The model was applied to the eye movement data from trials with natural scene background stimuli (Fig. S3). The main results remain unchanged from the results shown in the main article obtained from the free viewing on gray background stimuli. The most noticeable difference is that GoF is generally low for natural scene background trials compared to the results from gray background trials. This is most likely because of an increase in the number of background fixations induced by the complex background images. This increase is indicated by the value of p_{BG}; it is increased from 0.23 to 0.41 for monkey H, and from 0.21 to 0.51 for monkey S. Occurrence of more background fixations causes larger variability in the numbers of intra-object and trans-object saccades across trials, and hence should degrade the performance of the model. Furthermore, the ratios of the saccade types other than intra-object and trans-object are not as constant as observed for the free viewing on gray background stimuli. This feature further degrades the performance of the model, because the model cannot account for such changes by construction.

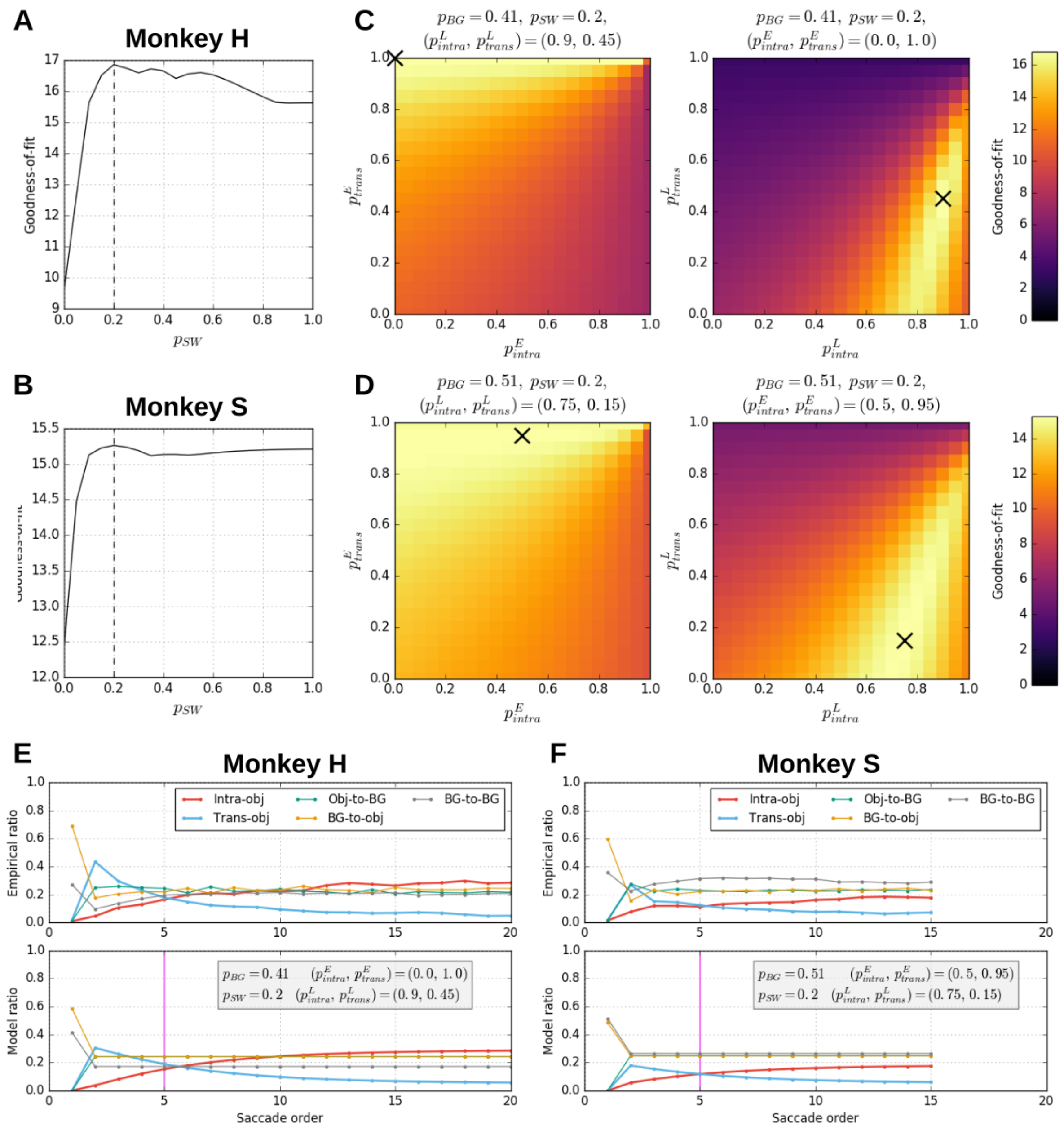

Supplementary Figure 3. Dependence of the goodness-of-fit (GoF) measure on model parameters. (A) Dependence of the GoF measure on the switching probability p_{SW} obtained from the fitting of the model to the data of monkey H from trials with natural scene background stimuli. The maximum GoF value achieved for each given p_{SW} value is plotted as a function of p_{SW}. Note that the values of the other parameters than p_{SW} are varied for different p_{switch} values so that the GoF is maximized under the constraint of each given fixed p_{switch} value. The value of p_{SW} is changed in steps of 0.05. The (unconstrained) maximum GoF value is taken at $p_{SW} = 0.20$, as indicated by the vertical dashed line. (B) Same as (A), but for monkey S. The maximum GoF value is taken at $p_{SW} = 0.25$. (C) Dependence of the GoF measure on the transition probabilities in the early mode (p^E_{intra} and p^E_{trans}; left) and those in the late mode (p^L_{intra} and p^L_{trans}; right), obtained from the fitting of the model to the data of monkey H. The other parameter values (indicated above the panels) are fixed so that the maximum GoF value (indicated by the black crosses) is contained in the respective plots. GoF values are represented by colors as indicated by the

color bar to the far-right. (D) Same as (C), but for monkey S. (E) Dependence of saccade type ratios on saccade order. Top: empirical ratios computed from the data of monkey H. Bottom: simulated ratios by the model with the best fitting parameter values (see Methods for how they are obtained), which are indicated in the gray box. The magenta vertical line in the bottom panel indicates the expected mode-switch timing derived as the inverse of p_{SW}. (F) Same as (E), but for monkey S. Note that, differently from Fig. 6E and F in the main article, the same initial condition (the initial fixation on the background) was used for simulations of monkey H and monkey S, because no object is placed at the center of the natural scene background stimuli used for the both monkeys.

Mathematical formulation of the model

Our stochastic model of saccade sequence generation described in the main article can be formalized as a hidden Markov model with four hidden states and three observations. The four states are identical to the saccade generation states shown in Fig. 5A of the main article, which we denote here as $\{I^E, T^E, I^L, T^L\}$, with the following correspondence: I^E and T^E are the intra- and trans-state of the early mode, respectively, and I^L and T^L are the intra- and the trans-state of the late mode, respectively. The three observations, which we denote as $\{O^S, O^D, BG\}$, are interpreted as the types of simulated fixations as follows: O^S is a fixation on the same object as the previous fixation was on, O^D is a fixation on an object different from the one that the previous fixation was on, and BG is a fixation on the background.

The state transition probabilities are defined as follows.

		To			
		I^E	T^E	I^L	T^L
From	I^E	$(1-p_{SW}) \cdot p^E_{intra}$	$(1-p_{SW}) \cdot (1-p^E_{intra})$	$p_{SW} \cdot p^L_{intra}$	$p_{SW} \cdot \left(1-p^L_{intra}\right)$
	T^E	$(1-p_{SW}) \cdot (1-p^E_{trans})$	$(1-p_{SW}) \cdot p^E_{trans}$	$p_{SW} \cdot (1-p^L_{trans})$	$p_{SW} \cdot p^L_{trans}$
	I^L	0	0	p^L_{intra}	$1-p^L_{intra}$
	T^L	0	0	$1-p^L_{trans}$	p^L_{trans}

The emission probabilities, which relate the current state of the model to the current observation, are defined as follows.

		Observation		
		O^S	O^D	BG
State	I^E	$1-p_{BG}$	0	p_{BG}
	T^E	0	$1-p_{BG}$	p_{BG}
	I^L	$1-p_{BG}$	0	p_{BG}
	T^L	0	$1-p_{BG}$	p_{BG}

Note that p_{SW}, p^E_{intra}, p^E_{trans}, p^L_{intra}, p^L_{trans} and p_{BG} are the same probabilities as those defined in the Results section and the Methods section of the main article.

The start probabilities, which specifies which state is taken at the initial time step of a simulation, for the four states are defined as $\{(1-p_{trans}^{E})/\left(2-p_{intra}^{E}-p_{trans}^{E}\right), \left(1-p_{intra}^{E}\right)/\left(2-p_{intra}^{E}-p_{trans}^{E}\right), 0, 0\}$ for $\{I^E, T^E, I^L, T^L\}$, respectively.

The above definitions give a complete specification of a hidden Markov model, which is equivalent to the model described in the main article. A sequence of observations generated by this model can be uniquely transformed to a sequence of simulated saccades by assigning a saccade type to each pair of successive observations as follows.

		Current		
		O^S	O^D	BG
Previous	O^S	Intra-object	Trans-object	Object-to-background
	O^D	Intro-object	Trans-object	Object-to-background
	BG	Background-to-object	Background-to-object	Background-to-background
